# Validation of a SNP-based non-invasive prenatal test to detect the fetal 22q11.2 deletion in maternal plasma samples

**DOI:** 10.1371/journal.pone.0193476

**Published:** 2018-02-23

**Authors:** Harini Ravi, Gabriel McNeill, Shruti Goel, Steven D. Meltzer, Nathan Hunkapiller, Allison Ryan, Brynn Levy, Zachary P. Demko

**Affiliations:** 1 Natera, Inc., San Carlos, CA, United States of America; 2 The Woman’s Hospital of Texas, Houston, TX, United States of America; 3 Department of Pathology and Cell Biology, Columbia University, New York, NY, United States of America; Hospital Authority, CHINA

## Abstract

**Introduction:**

Non-invasive prenatal testing (NIPT) for aneuploidy using cell-free DNA in maternal plasma has been widely adopted. Recently, NIPT coverage has expanded to detect subchromosomal abnormalities including the 22q11.2 deletion. Validation of a SNP-based NIPT for detection of 22q11.2 deletions demonstrating a high sensitivity (97.8%) and specificity (99.75%) has been reported. We sought to further demonstrate the performance of a revised version of the test in a larger set of pregnancy plasma samples.

**Methods:**

Blood samples from pregnant women (10 with 22q11.2-deletion‒affected fetuses and 390 negative controls) were successfully analyzed using a revised SNP-based NIPT for the 22q11.2 deletion. The sensitivity and specificity of the assay were measured.

**Results:**

Sensitivity of the assay was 90% (9/10), and specificity of the assay was 99.74% (389/390), with a corresponding false positive-rate of 0.26%.

**Discussion:**

The data presented in this study add to the growing body of evidence demonstrating the ability of the SNP-based NIPT to detect 22q11.2 deletions with high sensitivity and specificity.

## Introduction

Non-invasive prenatal testing (NIPT) using cell-free DNA from maternal plasma has altered the landscape of prenatal screening, owing to its improved performance over traditional screening techniques [1, 2]. Clinical adoption of prenatal screening for commonly observed aneuploidies has been rapid since its introduction in 2011 [2]. Recently, NIPT coverage has expanded to include a range of sub-chromosomal abnormalities with high penetrance and severe phenotypes, including the 22q11.2 deletion syndrome (22q11.2 DS also known as DiGeorge or velocardiofacial syndrome) [3–5].

With a reported population-wide frequency of 1 in 3,000–6,000 live births, 22q11.2 DS is the most common microdeletion syndrome [6, 7]. Several reports suggest a higher prenatal prevalence, with an estimated frequency of approximately 1 in 1000 [4, 8, 9]. 22q11.2 DS is characterized by a spectrum of clinical manifestations with varying degrees of severity, including congenital heart defects, immune dysfunction, hypocalcemia, mild-to-severe learning disabilities, and an increased risk of mental health disorders in adulthood [10]. Due to the variability in presentation, diagnosis based on clinical findings is challenging, and 22q11.2 DS remains undiagnosed in approximately one third of affected infants. For those cases diagnosed prenatally, appropriate prenatal counselling addressing the variable phenotype of the condition offers expectant couples the opportunity to make informed reproductive decisions and plan for prenatal and neonatal patient management. Patients who do not receive a perinatal diagnosis often undergo a lengthy process of evaluation by multiple specialist providers before a definitive diagnosis by molecular cytogenetics is reached [10].

For individuals with 22q11.2 DS pregnancy, changes in management such as delivery at specialized neonatal care centers may be advised and early therapeutic intervention can positively affect clinical outcomes [11]. For example, the majority (~87%) of deaths associated with the syndrome are attributed to congenital heart defects, [10] but appropriate care at birth can reduce related mortality by ~12% [12]. Likewise, hypocalcemia-induced seizures can be optimally managed by close monitoring of calcium levels and providing calcium supplementation as needed [13, 14]. Currently there is a paucity of information about the phenotype for 22q11.2 deletion cases that are ascertained through NIPT and how detection translates into improved long-term outcomes.

Previously, validation of a targeted single–nucleotide-polymorphism (SNP)-based test targeting the 22q11.2 deletion (~3 Mb A–D region) demonstrated an analytical detection rate of 97.8% and a false positive rate of 0.76% [8]. In that study, only three affected pregnancy plasma samples were analyzed along with 43 artificially generated mixture samples. The paucity of pregnancy plasma samples was considered a key limitation of the study [15]. Herein, we present results from an analytical validation of a revised version of the 22q11.2 deletion assay in a cohort consisting entirely of pregnancy plasma samples.

## Methods

### Ethics statement

Pregnant women were enrolled at six collaborating prenatal centers and all samples were collected in accordance with the following institutional review board (IRB)-approved protocols: Columbia University IRB (Columbia University, New York, NY), The Children's Hospital of Philadelphia Research Institute IRB Children's Hospital of Philadelphia, PA), Western IRB (Saint Peter's University Hospital, New Brunswick, NJ; GenoMed, Inc. Leesburg, FL; Advanced Bioscience Resource, Alameda, CA) and Biomed IRB (StemExpress, Placerville, CA). A signed informed consent was obtained from all enrolled participants.

### Study samples

Two tubes of blood (approximately 20 ml) were obtained from study participants who underwent amniocentesis or chorionic villus sampling; the study groups consisted of 10 women with a confirmed molecular diagnosis of fetal 22q11.2 deletion (nine women initially ascertained through ultrasound abnormalities often associated with 22q11.2DS; one woman had no prior risk indications associated with the disorder) and 409 women with a confirmed unaffected pregnancy based on molecular analysis (with no ultrasound findings suggestive of the disorder). For each sample, information regarding maternal age and gestational age of the patient at the time of the blood draw, and time between invasive procedure and blood draw was requested. Out-of-specification samples (i.e., gestational age <9 weeks, blood draw for NIPT performed post-fetal demise, samples with smaller nested proximal or atypical distal deletions (~13% of 22q11.2 deletions [8, 16], or inability to determine truth from an invasive-testing sample were excluded from the study analysis. A second sample was not requested for cases with no NIPT results. All cases were de-identified prior to NIPT analysis. The study was internally blinded; blinding with was overseen by one of our academic collaborators (BL) who served as an auditor.

### Single–nucleotide-polymorphism-based analyses

Maternal plasma samples were analyzed at Natera’s Clinical Laboratory Improvement Act-certified, and College of American Pathologists-accredited laboratory using an updated SNP-based screening methodology. The protocol uses a single set of pooled targeted primers to perform a massively multiplexed PCR (mmPCR) amplification targeting SNPs covering chromosomes 13, 18, 21, 22, X, and Y. The target set contains 13,926 distinct genetic loci, including 1,351 SNPs spanning a 2.91 Mb section of the 22q11.2 region that constitutes ~87% of all deletions detected in individuals with the 22q11.2 DS. Following amplification, samples were sequenced using the NextSeq 500 platform (Illumina, Inc., San Diego, CA) at a standard depth of read (~ 4.7x10^6^ reads/sample), and SNP allele ratios were determined (Panorama Precision Genomics SaaS Platform, V 2.0.12.0) to generate a fetal copy number for the 22q11.2 region. Risk status for the 22q11.2 deletion was assigned as “high risk” (≥0.95 confidence for the presence of the 22q11.2 deletion), “low risk” (≥0.90 confidence for the absence of the deletion), or “risk unchanged”/”no calls” (lower confidence values for both the presence and absence of the deletion) [8]. “High-risk” calls with maternally deleted haplotypes were reflexively sequenced at a higher depth of read (~ 14x10^6^ reads/sample) to confirm “high-risk” status. For cases with a fetal fraction of 2.8–6.5%, the sample was evaluated only for the presence or absence of the paternally-inherited haplotype.

### Data analysis

Comparison of patient demographics between the unaffected and affected groups was performed using independent samples t-tests for the difference between the means; p-values were 2-sided, and the significance level was set at 0.05. Sensitivity and specificity of the test to detect the 22q11.2 deletion were determined after reflex resequencing. Sensitivity was calculated as the proportion of cases with the 22q11.2 deletion that were identified “high-risk” for the 22q11.2 deletion by NIPT. Specificity was calculated as the proportion of truly unaffected cases that were assigned a “low-risk” status for the 22q11.2 deletion. Samples reported as “risk unchanged” or “no-call” were excluded from specificity calculations.

Based on a prevalence of 1/1442 for A–D deletions associated with 22q11.2 DS detected by the SNP-based test in the clinical population [16], an estimated positive predictive value (PPV) for the test was calculated by employing the standard formula, (sensitivity*prevalence)/ (sensitivity*prevalence + (1-specificity)*(1-prevalence)) [17], which uses estimated prevalence, sensitivity, and specificity.

## Results

To validate the performance of the updated SNP-based technology in detecting the 22q11.2 deletion, 419 pregnancy plasma samples with known fetal copy number for the 22q11.2 region were evaluated. The study included 10 samples with a confirmed affected fetus (Table 1) and 409 samples with an unaffected fetus for the 22q11.2 deletion, as determined by chromosomal microarray (5 affected samples), fluorescence *in situ* hybridization (FISH) (4 affected samples), or massively multiplexed PCR (mmPCR)/NGS analysis (1 affected, 409 unaffected samples) [8] on fetal genetic material obtained from an invasive procedure. For all samples, the mother was determined to be disomic based on analysis of the maternal circulating free DNA (cfDNA).

**Table 1:** Clinical information for affected samples.

No.	Deletion Syndrome	MA (Years)	GA (Weeks)	FF (%)	Procedure for Sample Procurement	DiagnosticTest	Deletion Size (Mb)	Time Between Invasive Test and Blood Draw (Days)	NIPT Call	Deleted Haplotype
1	22q11.2	N/A	22.0	13.3	Amnio	CMA	2.55	15	High risk	Paternal
2	22q11.2	19	15.6	11.7	CVS	mmPCR	≥2.91	0*	High risk	Paternal
3	22q11.2	20	30.4	15.4	Amnio	FISH	N/A	53	High risk	Maternal
4	22q11.2	21	21.5	39.7	Amnio	CMA	2.55	14	High risk	Maternal
5	22q11.2	35	13.3	19.4	CVS	FISH	N/A	6	High risk	Maternal
6	22q11.2	31	20.2	7.9	Amnio	CMA	2.55	0*	High risk	Maternal
7	22q11.2	35	25.1	8.0	Amnio	CMA	3.15	15	Low risk	N/A
8	22q11.2	30	16.2	21.4	Amnio	FISH	N/A	6	High risk	Maternal
9	22q11.2	31	14.0	9.6	CVS	FISH	N/A	2	High risk	Paternal
10	22q11.2	31	37.4	19.3	Amnio	CMA	2.55	109	High risk	Maternal

Amnio, amniocentesis; CMA, chromosomal microarray; CVS, chorionic villus sampling; FF, fetal fraction; FISH, fluorescence in situ hybridization; GA, gestational age; MA, maternal age; mmPCR, massively-multiplexed polymerase chain reaction; N/A, not available, NIPT, non-invasive prenatal testing.

*Blood draws were performed prior to procedures.

Among the 10 affected samples, the deletion was confirmed by array or mmPCR to occur in the common A–D region in six samples and deletion sizes ranged from 2.55–3.16 Mb. The remaining four affected samples were confirmed by FISH and were conservatively treated as having the full ~3 Mb A–D deletion (Table 1). In 8 of the 10 affected samples, the blood draw for NIPT was performed ≥2 days following the invasive procedure; in the other 2 cases, blood was drawn prior to the invasive procedure (Table 1). Of the 409 unaffected pregnancy plasma samples, 19 (4.6%) received a no-call due to low fetal fraction (fetal fraction <2.8%; n = 10), or inability of the algorithm to make a high confidence call (n = 9).

The mean maternal age for the affected samples was 28.1 weeks compared to 26.0 weeks for unaffected samples (p-value of 0.351). The gestational age range was higher (mean 21.7 weeks, range 13.4–37.6) for the affected samples compared to the unaffected samples (mean 12.8 weeks, range 9.0–27.0) with a p-value of 0.005. The affected samples also had a higher fetal fraction distribution (mean 16.6%, 7.9–39.7%) compared to the unaffected samples (mean 9.4%, 2.8–31.0%) with a p-value of 0.04 (Table 2).

**Table 2:** Patient characteristics of study samples.

Patient Characteristics	Affected Samples (n = 10)	Unaffected Samples (n = 390)	p-value
Maternal Age (Years)*^‡^			
Mean ± SD	28.1 ± 6.4	26.0 ± 5.3	0.351
Median (Range)	31.0 (19.0–35.0)	25 (18.0–40.0)	
Gestational Age (Weeks)*			
Mean ± SD	21.7 ± 7.7	12.8 ± 3.2	0.005
Median (Range)	21.0 (13.4–37.6)	12.4 (9.0–27.0)	
**Fetal Fraction (%)**			
Mean ± SD	16.6 ± 9.5	9.4 ± 4.2	0.04
Median (Range)	14.4 (7.9–39.7)	8.8 (2.8–31.0)	

*At date of blood draw.

^‡^Maternal age data were available for 9/10 affected samples.

Of the 10 affected pregnancy samples, 9 were identified as high-risk (test positive) and 1 was identified as low-risk (test negative), generating a sensitivity of 90% (9/10; 95% confidence interval, 55.5–99.75%) (Table 3). All high-risk calls were further evaluated for both paternal and maternal haplotypes to identify the parental source of the 22q11.2 deletion. Of the 9 samples that received a high-risk call, 6 were found to be deleted on the maternally inherited chromosome and 3 on the paternally inherited chromosome (Table 1). Of the 390 unaffected samples, one false positive case was reported (gestational age 15 weeks; fetal fraction 8.5%; no evidence of chromosomal anomalies in the CVS sample), generating an observed specificity of 99.74% (389/390; 95% confidence interval, 98.58%–99.99%) (Table 3) with a corresponding false positive rate of 0.26% (95% confidence interval, 0.01–1.42%). Low-risk calls for 99 unaffected cases with fetal fraction ranging from 2.8–6.5% were based on the analysis of only the paternal allele. Based on a prevalence of 1/1442 in the clinical pregnancy population for the A–D 22q11.2 deletion [16], the estimated PPV is 19.6% (Table 3).

**Table 3:** Sensitivity, specificity, and estimated positive predictive value for 22q11.2 A–D deletions.

**Sensitivity, % (n) (95% CI)**	90.0 (9/10) (55.50–99.75)
**Specificity, % (n) (95% CI)**	99.74 (389/390) (98.58–99.99)
**Prevalence**	1/1442 [16]
Estimated Positive Predictive Value*, %	19.6

*Calculated by the standard formula for estimating PPV based on prevalence, sensitivity, and specificity

## Discussion

Previously, we reported that the SNP-based NIPT detects the fetal 22q11.2 deletion (~3 Mb A–D deletion) with a sensitivity of 97.8% and a false positive rate of 0.76% [8]. To reduce the false positive rate and lower the barrier to clinical adoption, the assay was revised in three ways. First, the number of targeted SNPs in the 22q11.2 region was increased from 672 to 1,351. Second, the algorithm’s positive-call confidence threshold was raised from 0.90 to 0.95 [16]. Finally, reflex sequencing of “high-risk” calls with maternally-deleted haplotypes at higher depth of read was implemented [4]. Data from this validation of the revised test on 400 pregnancy plasma samples (which included 10 affected samples) demonstrated a sensitivity of 90% and a false positive rate of 0.26%, in line with expected assay performance given the methodological improvements and previous reports [8, 16]. Of note, this SNP-based test is designed to detect the large A–D 22q11.2 deletion that is associated with 22q11.2 DS[8, 16]. Performance of the assay has not yet been assessed for smaller deletions within the A-D region. Thus, a conservative estimate of the detection rate under the assumption that none of these smaller deletions are identifiable would be approximately 78.3% [the proportion of all 22q11.2 DS that consist of full A–D deletions (87%) x the measured sensitivity of the assay for the full A–D deletions (90%)]. Based on a prenatal test referral prevalence of 1/1442 for full A–D deletions (or 1/1225 for all significant deletions in A–D deletions) the PPV was 19.6%. Alternately, using a population-wide prevalence number of 1/4,000 [6, 7], the estimated PPV of the assay would be 8.1%. The PPV of this assay was directly measured in a clinical population previously and was found to be 44.2% [16].

The false positive rate with the revised methodology was 0.26% compared with 0.76% in the previous validation study [8]. However, precise measurement of a false positive rate close to zero requires a very large study cohort. A clinical experience study reporting results in more than 70,000 samples tested for fetal 22q11.2 deletion yielded an estimated false positive rate of 0.07% when using the same revised protocol [16]. Although clinical follow-up was not available for all women in that study, an estimate of the false positive rate was made using the conservative assumption that all “high-risk” cases lost to follow-up were false positives, yielding an upper-bound false positive rate estimate of 0.09% and a corresponding specificity of 99.91%. Caution should be exercised, however, when interpreting false positive rates—false positive cases may harbor smaller deletions in 22q11.2 that could remain undetected by FISH, which is often used as the confirmatory test for subchromosomal imbalances. Confined placental mosaicism might also contribute to the false positive rate [18].

A limitation of this study is the higher gestational age distribution of the affected pregnancy cases compared with those typically observed in a clinical cohort. The higher gestational age is attributed to the delay in identifying and procuring samples from affected pregnancies. As gestational age and fetal fraction are positively correlated, higher fetal fractions among the affected pregnancies would be expected to affect the assay’s performance. Note that in our previous validation study, which used mixture samples, the fetal fraction distribution of the affected samples was specifically designed to approximate that of a clinical cohort [8], and in that study, the sensitivity was comparable to the sensitivity observed in this study. Another factor that may affect fetal fraction is the time between the invasive procedure (CVS or amniocentesis) and the blood draw for NIPT. Although it has been suggested that fetal fraction may rise immediately following an invasive procedure [19], another study reported no evidence of an increase in fetal fraction 24 hours following a CVS procedure [20]. All blood samples in this study were either drawn prior to, or >24 hours after invasive procedures.

The relative rarity of the 22q11.2 deletion makes it challenging to assemble a patient cohort closely resembling a clinical population for use in a validation study. Historically, validation studies for rare conditions required alternative approaches—e.g., testing maternal plasma diluted with DNA from an affected individual [8, 21]. As NIPT coverage expands to include rarer conditions, indication-specific analytic validations using affected pregnancy plasma samples, as obtained for this study, will prove increasingly difficult to do, and alternative validation approaches will become necessary. It is therefore notable that in this study, consisting entirely of pregnancy plasma samples, the demonstrated assay performance was comparable to that reported in a previous study that used a substantial number of simulated pregnancy samples [8]. Another recent study reported the performance of a microarray-based cfDNA test for the detection of the 22q11.2 deletion that included approximately 120 affected lab-generated pregnancy plasma samples [22], further supporting the use of laboratory-generated samples as substitutes for clinical samples.

Subchromosomal abnormalities occur in >1% of pregnancies and are often associated with severe phenotypes [23]. NIPT has recently expanded its coverage of testing to include a subset of clinically significant microdeletions. The 22q11.2 DS is a well-defined, severe condition, and improved clinical outcomes have been demonstrated with prenatal diagnosis [10]. The data presented in this study add to the growing body of evidence demonstrating the ability of the SNP-based NIPT to detect 22q11.2 deletions with high sensitivity and specificity [4, 8].
